# Epidemiology of echinococcosis in Iran: a systematic review and meta-analysis

**DOI:** 10.1186/s12879-019-4458-5

**Published:** 2019-11-04

**Authors:** Shima Mahmoudi, Setareh Mamishi, Maryam Banar, Babak Pourakbari, Hossein Keshavarz

**Affiliations:** 1 Pediatric Infectious Disease Research Center, Tehran University of Medical Sciences, Tehran, Iran; 2 Center for Research of Endemic Parasites of Iran (CREPI), Tehran University of Medical Sciences, Tehran, Iran; 3 Department of Medical Parasitology and Mycology, School of Public Heath, Tehran University of Medical Sciences, Poursina st, P.O. Box 14155-6446, Tehran, Iran

**Keywords:** Cystic echinococcosis, Alveolar echinococcosis, Prevalence, Iran

## Abstract

**Background:**

Echinococcosis is a zoonotic disease caused by the larval stages of taeniid cestodes of the genus *Echinococcus*. The two major types of infection in humans are cystic echinococcosis (CE) or hydatidosis and alveolar echinococcosis (AE). It is endemic in some parts of the world, such as the Middle East, with Iran being a part of it. This systematic review and meta-analysis were conducted to determine the prevalence of CE and AE echinococcosis and their epidemiological and clinical aspects in Iran.

**Methods:**

Electronic databases, including MEDLINE (via PubMed), SCOPUS, Web of Science, SID and Mag Iran (two Persian scientific search engines) were searched from 1 January 1990 to 8 August 2017. The prevalence of CE and AE echinococcosis was estimated using the random effects meta-analysis. Heterogeneity was evaluated by subgroup analysis. Data were analyzed by STATA version 12.

**Results:**

Of the 2051 records identified in the mentioned electronic databases, Seventy-eight articles met our eligibility criteria, with a total of 214124individuals. The meta-analysis was performed on only 37 out of 78 included studies. The pooled prevalence of CE and AE in Iran was 5% [95% confidence interval )CI(: 3-6%] and 2% [95% CI: 0-5%], respectively. Subgroup meta-analysis revealed that the prevalence of CE was significantly higher in North [9%, 95% CI: 4-18%] and West of Iran [6%, 95% CI: 3-11%], patients younger than 40 years of age [7%, 95% CI: 4-12%], villagers and nomads [6%, 95% CI: 2-12%], and studies that used the combination of serological, clinical, and imaging diagnostic methods [7%, 95% CI: 5-9%]. There were no significant differences between the prevalence of CE among low and high-quality studies. Housewives were the most affected group by hydatidosis (*n*=24/77, 31%), followed by illiterate people (*n*=11/77, 14%) and farmers (*n*= 9/77, 12%). Liver [55%, 95% CI: 46-65%] and lung [28%, 95% CI, 22-35%] were the most common sites of cyst formation.

**Conclusions:**

Given to the importance of echinococcosis on human health and domestic animals industry, it is necessary to implement monitoring and control measures in this regard.

## Background

Echinococcosis is an important chronic zoonotic disease in many parts of the world including Iran [1–3], which caused by the larval stages of the parasites belonging to the genus *Echinococcus* [4, 5]. According to the estimation of World Health Organization (WHO), more than one million people are affected by echinococcosis globally each year and in 2015, resulted in 19300 death cases around the world. Every year, the cost of treating patients with echinococcosis as well as damage to the livestock industry is about 3 billion dollars [6].

Humans are accidental intermediate hosts and acquire the infection via direct contact with infected final hosts (i.e. carnivores such as dogs, foxes, coyotes, and wolves) or via ingestion of parasite’s embryonated eggs in contaminated food, water, or soil [1, 6, 7]. The two major types of infection in humans are cystic echinococcosis (CE) or hydatidosis (caused by the species *E. granulosus*) and alveolar echinococcosis (AE) (caused by the species *E. multilocularis*) that have serious health and economic implications and vast geographical distribution [5, 6, 8]. CE is characterized by the formation of one or more cystic lesions in the liver, lung, kidney or other body organs and AE is defined as the development of a tumor-like lesion that usually occurs in the liver [4, 6].

According to the previous systematic review and meta-analysis study conducted by Shafiei *et al*., in 2016 [2], the estimated prevalence of CE in Iran was 5 % [95% CI: 4-7%]. The disease was most frequent among women and older patients, and the highest prevalence of CE was observed in the southwest and south of Iran. In another systematic review and meta-analysis done by Khalkhali *et al*., in 2017 [9], the prevalence of human hydatidosis was 4.2% [95% CI: 3-5.5%]. Most cases were women, and the disease was most prevalent in rural regions and southern Iran.

Studies demonstrated that the most prevalent genotypes of *E. granulosus* in Iran are G1 (92.75%) and G6 (4.53%) in sheep, cattle, camels, goats, and buffaloes [10]. G1 is also the most frequent genotype in human CE [10, 11].

Every year a lot of body organs (especially livers and lungs) are damaged due to the infection with this parasite and imposes considerable costs on the health care system [12]. The expense of human CE in Iran was estimated at US$93.39 million [95% CI US$6.1–222.7 million]. In addition, the cost of surgery for each human case of CE was estimated at US$1,539 [13].

With regard to the medical and economic importance of this disease, it is essential to implement strong monitoring programs in order to assess the burden of disease and the progress made in control programs [6]. We accomplished a systematic review and meta-analysis to determine the prevalence of CE and AE echinococcosis and their epidemiological and clinical aspects in Iran.

## Methods

### Search method and selection criteria

Three English electronic databases (MEDLINE (via PubMed), SCOPUS, and Web of Science) and two Persian electronic databases (Magiran and Scientific Information Database (SID)) were searched systematically from 1 January 1990 to 8 August 2017. Publication searches were performed by various combinations of the following terms: “Hydatidosis” or “Echinococcosis” or “Hydatid cyst” or “*Echinococcus granulosus*” or “*Echinococcus multilocularis*” or “Alveolar hydatid cyst” or “liver and alveolar hydatid cyst” or “hepatic alveolar hydatid cyst” or “Alveolar echinococcosis” or “Cystic echinococcosis” AND “Human” AND “Iran”. The reference lists of selected articles also were screened manually and appropriate articles were included. Abstracts of papers published in congresses were not reviewed because they did not have enough details for quality assessment. Dissertations and thesis were not included. The study was conducted according to the guidelines of PRISMA (the preferred reporting items for systematic reviews and meta-analyses) [14]. Titles and abstracts of all articles were screened by one reviewer, and eligibility of the screened articles was assessed by two independent investigators using the following criteria.

#### Inclusion criteria

##### Case definition

A patient was diagnosed as echinococcosis-infected case if his blood sample was positive for anti-echinococcus antibodies (diagnosed by enzyme-linked immunosorbent assay (ELISA), or indirect hemagglutination antibody test (IHA), or indirect fluorescent antibody test (IFA), or counterimmunoelectrophoresis (CIE)) or his clinical characteristics (diagnosed by computed tomography (CT) scan, magnetic resonance imaging (MRI), chest-X ray, ultrasonography, bronchoscopy, or radiology) or pathological properties were indicative of echinococcosis [15].

Articles were included whether they have recognized echinococcosis based on clinical symptoms /imaging or radiographic characteristics or identified the disease using serological tests or pathological examinations. Only articles that reported human echinococcosis were included. Iranian papers in each language of Persian or English were enrolled.

#### Exclusion criteria

Investigations with not-relevant topics, review and case report articles were excluded. Reports from other countries were disqualified.

### Data extraction

Two independent reviewers read the included articles in full text and extracted the following data: first author’s name, year of study, location of study, sample size, gender of patients, the most affected age group, number of patients diagnosed with echinococcosis, types of echinococcosis (i.e. CE or AE), diagnostic methods used, and the most common infected group. Any disagreement between the two reviewers was resolved by consensus.

### Quality assessment

The quality of the included articles was assessed using the Joanna Briggs Institute (JBI) critical appraisal checklist for studies reporting prevalence data [16]. This action was performed by two independent reviewers and any dispute was resolved through discussion.

### Meta-analysis

Random-effects model was used to pool the estimations; and standard methods recommended for meta-analyses of prevalence were employed. Outcomes were the total prevalence of CE and AE in Iran and the prevalence of CE in different sites of body. Results of the meta-analysis were shown as a forest plot diagram, which represents the estimated prevalence and their relevant 95% confidence interval (CI). The Cochran’s heterogeneity statistic (푄-test) and 퐼^2^ statistic were used to examine the heterogeneity of studies. The *I*^*2*^ values of 25%, 50%, and 75% were considered as low, medium, and high heterogeneity, respectively.

Subgroup meta-analysis was utilized to compare the prevalence of CE on the basis of geographic distribution of studies (North, Center, West, and East of Iran), patients’ age (age < 4o years vs. age ≥ 40 years), quality score of the studies (the study score <5 (low quality) vs. the study score ≥ 5 (high quality)), diagnostic lab methods used in the studies (serological vs. serological and clinical methods), and place of residence of the patients (urban, urban and rural, rural and nomad). The Q and *I*^*2*^ statistics values were calculated for each subgroup to determine the effective factors on the prevalence of CE and heterogeneity of the studies. Publication bias were evaluated by Egger’s regression test [17].

This meta-analysis procedure was accomplished using STATA software (Release 12. statistical software. College Station, Texas: STATA Corp LP).

### Study area

Iran consists of a land area of over 1.6 million square kilometers. It is bounded by Iraq and Turkey in the west, Afghanistan and Pakistan in the east, the Persian Gulf and Oman Sea in the south, as well as Caspian Sea, Azerbaijan, Armenia and Turkmenistan in the north. The country generally features three climatic zones, including: arid and semi-arid climate of the interior and far south, mountainous climate, and Caspian climate. In the year 2012, Iran was divided into 31 provinces [18] and according to the last Population and Housing Census taken in 2016; the total population of the country was 79926270 persons (comprising 50.66% male and 49.34% female), which 59146847 persons (74%) were urban dwellers and the 20730625 persons (26%) were settled in rural areas [19].

## Results

**Fig. 1:**
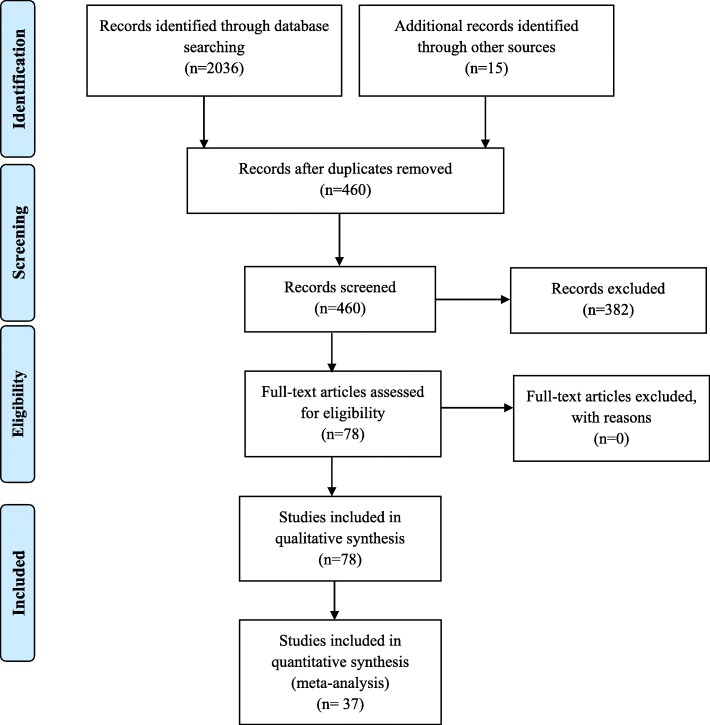
Summary of the literature search and study selection

Of the 2051 records identified in the mentioned electronic databases and through articles’ reference lists (Fig. 1), 78 articles met inclusion criteria and enrolled into the systematic review. Only 37 articles that reported the prevalence of CE and AE in their understudy populations were included in the meta-analysis. The characteristics of the included studies are summarized in Tables 1 and 2.

**Table 1:** Characteristics of the studies that reported cystic echinococcosis (CE) in Iran

	First author (year of study)	Province	Sample size	Male Sex N (%)	The most affected age group (year)	Positive echinococcosis *N* (%)	Diagnostic Method^b^	The most common infected group (%)	Total qualityscore
1	Aslanabadi (2001-2011) [20]	Ardabil / East Azerbaijan	59	32 (54.2)	ND ^a^	59 (100)	CT scan	ND	5
2	Mirzanejadasl (2008) [21]	Ardabil	1998	14 (7.9)	40-59	184 (9.2)	ELISA	ND	2
3	Mirzanejadasl (2010) [22]	Ardabil	1003	10 (11.2)	40-59	92 (9.2)	Ultrasonography, CT scan, ELISA	ND	4
4	Heidari (2011) [23]	Ardabil	670	4 (33.33)	60-90	12 (1.79)	ELISA	Farmers (3.17), Ranchmen (3.17), Illiterate people (2.6), consumption of unwashed raw vegetables (3.88)	7
5	Khalili (1998-2007) [24]	Chaharmahal and Bakhtiari	144	46 (32)	27-41	144 (100)	Clinical and laboratory findings	Rural dwellers (65), Housewives (60)	6
6	Yousefi Darani (2000-2001) [25]	Chaharmahal and Bakhtiari	2524	56 (4.4)	ND	120 (4.8)	CIE	ND	5
7	Montazeri (1995-2005) [26]	East Azerbaijan	383	207 (54)	ND	383 (100)	Chest X-ray	ND	4
8	Montazeri (1995-2005) [26]	East Azerbaijan	62	40 (64)	ND	62 (100)	Chest X-ray	ND	4
9	Vahedi (2001-2012) [27]	East Azerbaijan	318	135 (42.5)	20-30	318 (100)	ND	ND	2
10	Ghabouli Mehrabani (2009-2011) [28]	East-Azerbaijan	52	25 (48)	40-60	52 (100)	ND	Rural dwellers (63), Housewives (44.2%),	6
11	Garedaghi (2011) [29]	East Azerbaijan	1500	5 (0.83)	30-40	19 (1.28)	ELISA	Self-employed people (2.8)	8
12	Dadkhah (2011) [30]	East Azerbaijan	250	3 (30)	20-40	8 (3.2)	IFA	Housewives, Illiterate people	3
13	Sadjjadi (1978-1998) [31]	Fars	1227	ND	ND	1227 (100)	Pathology, CCIEP, Ultrasound	ND	1
14	Saberi-Firouzi (1994- 1995) [32]	Fars	1000	20 (4.5)	15 - 29	50 (5)	Ultrasonography, ELISA, CIE	Illiterate people (5), Carpet weaver (5.3), contact with dog (5.4)	7
15	Solhjou (2006-2007) [33]	Fars	1096	45 (65.2)	46-60	69 (6.3)	ELISA	Farmers (18.18), Illiterate people (8.18)	8
16	Sarkari (2013-2014) [34]	Fars	1068	56 (5.5)	> 50	60 (5.6)	ELISA	Self-employed people (46)	5
17	Aghajanzadeh (1992-2005) [3]	Guilan	152	ND	ND	152 (100)	Chest X-ray, CT scan, Ultrasonography	ND	0
18	Mansour-Ghanaei (2000-2010) [35]	Guilan	62	38 (61)	40-60	62 (100)	Ultrasonography, CT scan, Chest X-ray, Pathology	Urban dwellers (66.1), Housewives (40.3),	6
19	Baharsefat (2007) [36]	Golestan	1024	20 (1.93)	40-49	46 (4.5)	IFA, ELISA	Housewives (3.67), Illiterate people (3.72),	5
20	Arbabi (1991)	Hamedan	1530	19 (2.9)	60-80	46 (3)	IFA, IHA	ND	8
21	Ahmadi (1992-2006) [37]	Hamedan	179	79 (44.1)	20-39	179 (100)	Ultrasonography, CT scan	Housewives (47.3), Rural dwellers (57.5)	8
22	Ahmadi (1992-2006) [38]	Hamedan	24	10 (42)	40–59	24 (100)	Ultrasonography, CT scan	Housewives (50), Rural dwellers (54.2)	6
23	Aflaki (2005) [39]	Ilam	3000	15 (1)	20-30	37 (1.2)	Dot-ELISA/ ELISA	Nomads (10.77)	4
24	Arbabi (1993-2000) [40]	Isfahan	85	38 (44.7)	20-29	85 (100)	ND	Housewives (47), Urban dwellers (68.2)	7
25	Arbabi (2006) [40]	Isfahan	500	4 (0.9)	< 20	12 (2.4)	IHA	Housewives (5)	7
26	Fahimzad (2001-2013) [41]	Isfahan, Tehran, Sistan and Baluchestan, Kurdistan, Kermanshah, Hamedan, Markazi	161	99 (61.5)	6-10 years	161 (100)	Pathology, Ultrasonography, Chest X-ray, CT scan,	ND	4
27	Esmaeili (2010) [40]	Isfahan	361	4 (2.3)	31-45	11 (3.05)	ELISA, IFA	Illiterate people (4.3), Housewives (6)	7
28	Ilbeigi (2015) [40]	Isfahan	635	14 (2.24)	60-69	7 (1.1)	ELISA	self-employed people (3.05)	4
29	Eftekhari (1991-2000) [42]	Kerman	60	31 (51.6)	21-30	60 (100)	Radiography, CT scan, Ultrasonography	Housewives (69), Rural dwellers (63.3)	6
30	Harandi (2006–2008) [43]	Kerman	1062	22 (2.1)	20-39	77 (7.3)	Ultrasonography, ELISA	Housewives (8.4)	7
31	Moazezi (2008-2009) [44]	Kerman	451	4 (4.9)	30-55	37 (8.8)	Ultrasonography, ELISA	Housewives (9.6), Farmers (9.1)	7
32	Vejdani (2004-2009) [45]	Kermanshah	526	141 (48.5)	31-40	291 (55)	CT scan, MRI, Ultrasonography, Pathology	Urban dwellers (57.7)	4
33	Chalechale (2009-2011) [46]	Kermanshah	172	79 (46)	21-40	172 (100)	ND	ND	3
34	Bagheri (1981–2008) [47]	Khorasan Razavi	1024	535 (52.2)	ND	1024 (100)	IHA, ChestX-ray, CT scan, Bronchoscopy, Ultrasonography	Housewives (27.2)	7
35	Ebrahimipour (2001-2008) [48]	Khorasan Razavi	400	218 (54.5)	20–29	400 (100)	MRI, Ultrasonography, IHA, ELISA,	ND	4
36	Andalib Aliabadi (2003-2012) [49]	Khorasan Razavi	1342	631 (47)	20-30	1342 (100)	Parasitology, Histopathology	Housewives, Urban dwellers (71)	6
37	Sadrizadeh (2010-2012) [50]	Khorasan Razavi	87	46 (52)	20-30	87 (100)	Chest X-ray, CT scan, IFA	ND	5
38	Khazaei (2011-2014) [51]	Khorasan Razavi	357	161 (45.1)	21-40	357 (100)	Radiology, CT scan	Housewives (45.8), Rural dwellers (40.3)	7
39	Fotoohi (2013) [52]	Khorasan Razavi	100	ND	ND	7 (0.07)	ELISA	ND	-2
40	Talaiezadeh (1994-2000) [53]	Khuzestan	40	24 (60)	6-10 years	40 (100)	Ultrasonography, Chest-X-Ray	ND	4
41	Rafiei (2007) [54]	Khuzestan	3446	176 (13.7)	0-4	475 (13.8)	ELISA	Farmers (18.5), Shepherds (15.8)	7
42	Sarkari (1997-2006) [55]	Kohkiluyeh and Buyer Ahmad	105	35 (33.33)	30-39	105 (100)	ND	Housewives (66.7)	6
43	Sarkari (2009) [56]	Kohkiluyeh and Buyer Ahmad	500	21 (58.3)	30-39	36 (7.1)	ELISA	Farmers	6
44	Akhlaghi (2005) [57]	Kurdistan	1114	925 (83)	20-40	81 (7.3)	IFA	Illiterate people	3
45	Hadadian (2006) [58]	Kurdistan	1979	5 (0.53)	30-40	22 (1.12)	ELISA	ND	7
46	Rostami Nejad (2002-2006) [59]	Lorestan	64513	17 (43.4)	ND	39 (0.06)	ND	Housewives (51)	6
47	Nayebzadeh (2004-2011) [60]	Lorestan	134	57 (42.5)	20-30	134 (100)	Pathology, CT scan, Ultrasonography, Radiology, Bronchoscopy	ND	4
48	Zibaei (2007) [61]	Lorestan	617	57 (60)	20-29	95 (15.4)	ELISA	Primary school educated (22.7), Farmers (59.3), Rural dwellers (38.9)	7
49	Zibaei (2007- 2011) [61]	Lorestan	58	28 (48.3)	20-29	58 (100)	ND	ND	7
50	Asgari (2013) [62]	Markazi	578	13 (2.31)	40-49	20 (3.46)	ELISA	Farmers (6.67), Ranchmen (6.67), Illiterate people (4.8), Rural dwellers (7)	7
51	Esfandiari (2001-2007) [63]	Mazandaran	132	59 (44.7)	21-30	132 (100)	ND	ND	2
52	Moosazadeh (2009-2014) [64]	Mazandaran	41	15 (36.6)	30-49	41 (100)	CT scan, MRI, Ultrasonography, Radiology	Housewives (58.5)	7
53	Ziaei Hezarjaribi (2013-2014) [65]	Mazandaran	600	148 (24.6)	30-60	190 (31.6)	ELISA	Rural dwellers (25.7), Educated people (25.8)	7
54	Salehi (2009-2011) [66]	North Khorasan	24	10 (41.7)	31-40	24 (100)	ND	Housewives (45.8)	5
55	Rezaei (2002-2013) [67]	Qom	82	35 (42.7)	21-30	82 (100)	ND	Illiterate people (62.2), Housewives (51.3)	6
56	Rakhshanpour (2011-2012) [68]	Qom	1564	18 (2.2)	30-60	25 (1.6)	ELISA	Employees (2.4), Urban dwellers (2.1)	7
57	Sharifi-Mood (1990-2005) [69]	Sistan and Baluchestan	49	37 (75.5)	ND	49 (100)	Chest X-ray, Chest roentgenogram, CT-Scan, ELISA,	Rural dwellers (81)	4
58	Moradi (2014) [70]	Sistan and Baluchestan	536	1 (0.18)	23	1 (0.18)	ELISA	self-employed people	7
59	Amini (1984-2004) [71]	Tehran	60	28 (46.7)	30-39	60 (100)	Ultrasonography, CT scan, Chest X-ray,	Housewives (75), Farmer (42.8)	6
60	Nourjah (1986-1990) [72]	Tehran	4850	1439 (43.6)	20-29	4850 (100)	ND	Housewives, Illiterate people (68.3)	5
61	Mirshemirani (1987-2007) [73]	Tehran	116	61 (52.6)	9-14 years	116 (100)	Radiography, CT scan, Ultrasonography, ELISA, IFA	ND	4
62	Mirshemirani (1992-2007) [74]	Tehran	72	40 (55.56)	12-14 years	72 (100)	Chest X- ray, ELISA, IFA, CT scan, Ultrasonography	ND	4
63	Pejhan (1995-2005) [75]	Tehran	82	48 (59)	ND	82 (100)	Chest X-ray, CT scan, Ultrasonography,	ND	4
64	Mamishi (1995-2005) [76]	Tehran	71,600	18 (58)	ND	31 (0.04)	Chest X-rays, CT scan, Ultrasonography	dwellers of central areas of Iran (58), Children with history of contact with dogs or sheep (52)	6
65	Khalilzadeh (1996-2004) [77]	Tehran	11	10 (91)	10-16 years	11 (100)	Chest X-ray, CT scan	ND	4
66	Mirshemirani (1996-2010) [78]	Tehran	100	54 (54)	13-14	100 (100)	Sonography, CT scan, ELISA	ND	5
67	Sedaghat Gohar (1999) [79]	Tehran	1052	12 (4.5)	> 60	62 (5.9)	IFA	Rural dwellers (8.1), Farmers and Ranchmen (16), Illiterate people (8)	7
68	Ahmadi (1999-2009) [80]	Tehran	203	86 (42)	21-40	203 (100)	Ultrasonography, CT scan, Chest X-ray, Endoscopy, Gastroscopy, MRI, IHA, ELISA, Casoni	Housewives (53.5), Urban dwellers (87)	7
69	Mousavi (2000-2010) [81]	Tehran	89	39 (44)	ND	89 (100)	Ultrasonography, CT scan	ND	4
70	Pezaeshki (2001-2004) [82]	Tehran	78	34 (43.5)	32-40	78 (100)	ND	ND	3
71	Mahmoudi (2005-2010) [83]	Tehran	17	9 (52.9)	ND	17 (100)	Ultrasonography, CT scan, IFA,	Inhabitants of central part of Iran (58.8),	4
72	Farrokhzad (2006) [84]	Tehran	437	ND	ND	1 (0.22)	IFA, ELISA	ND	1
73	Akhlaghi (2006-2007) [85]	Tehran	1100	ND	ND	18 (1.63)	Dot-ELISA	ND	0
74	Zarif-fard (1999) [86]	Western Iran	4138	11 (4.7)	21-40	230 (5.55)	ELISA	Kurd (6.1) and Lour (5.8) ethnic groups, Hunters (7.4)	6
75	Hajipirloo (2000-2009) [87]	West Azerbaijan	294	137 (46.7)	20-30	294 (100)	ND	Rural dwellers (63.1)	5
76	Fattahi Bafghi (2006-2011) [88]	Yazd	26911	8 (66.67)	8-28 and 49-69	12 (0.045)	ND	ND	3
77	Kohansal (2007-2013) [89]	Zanjan	136	62 (45.58)	21–40	136 (100)	ND	ND	3

^a^*ND* Not-determined. ^b^ *CT scan* computed tomography, *ELISA* Enzyme-linked immunosorbent assay, *CIE* Counterimmunoelectrophoresis, *IFA* Indirect fluorescent antibody test, *IHA* Indirect hemagglutination antibody test, *MRI* Magnetic resonance imaging, *CCIEP* Counter-current immunoelectrophoresis

**Table 2:** Characteristics of the studies that reported alveolar echinococcosis (AE) in Iran

	First author (year of study)	Province	Sample size	Male Sex N (%)	The most affected age group (year)	Positive N (%)	Diagnostic Method^b^	The most common infected group (%)	Total score
1	Mirshemirani (1987-2007) [73]	Tehran	116	ND ^a^	ND	3 (2.6)	Radiography, CT scan, Sonography, ELISA, IFA	ND	4
2	Mirshemirani (1992-2007) [74]	Tehran	72	ND	ND	1 (2)	Chest X- ray, ELISA, IFA, CT scan, Ultrasonography	ND	4
3	Maddah (1997-2012) [90]	Khorasan Razavi	18	4 (21)	ND	18 (100)	Ultrasonography, CT scan	ND	4

^a^*ND* Not-determined. ^b^ *CT scan* Computed tomography, *MRI* Magnetic resonance imaging, *ELISA* Enzyme-linked immunosorbent assay, *IFA* Indirect fluorescent antibody test

In overall, studies had a wide geographical distribution and were carried out in 25 different provinces of Iran. Most studies (15 articles) were from Tehran (*n*= 79867) [71–85], seven were from Khorasan Razavi (*n*=3328) [47–52, 90], six were from East Azerbaijan (*n*= 2565) [26–30], four each were from Isfahan (*n*=1581) [40, 91, 92] and Lorestan (65322) [59–61], three each were from Mazandaran (*n*=773) [63–65], Kerman (*n*=1573) [42–44], Ardabil (*n*=3671) [21–23], and Hamedan (*n*=1733) [37, 38, 93], two each were from Chaharmahal and Bakhtiari (*n*=2668) [24, 25], Fars (*n*=4391) [31–34], Guilan (214) [3, 35], Kermanshah (*n*=698) [45, 46], Khuzestan (*n*=3486) [53, 54], Kohkiluyeh and Buyer Ahmad (*n*= 605) [55, 56], Kurdistan (*n*=3093) [57, 58], Qom (*n*=1646) [67, 68], Sistan and Baluchestan (*n*=585) [69, 70], one each were from Golestan (*n*=1024) [36], Ilam (*n*=3000) [39], Markazi (*n*=578) [62], North Khorasan (*n*=24) [66], West Azerbaijan (*n*=294) [87], Yazd (*n*=26911) [88], and Zanjan (*n*=136) [89]. One study was performed in both Ardabil and East Azerbaijan (*n*=59) [20], one study reported the number of hydatidosis infected patients admitted to eight major referral hospitals in Isfahan, Tehran, Sistan and Baluchestan, Kurdistan, Kermanshah, Hamedan, and Markazi (*n*=161) [41], and finally, one study examined the serum samples of healthy volunteers from 8 different western provinces of Iran including Ardabil, East Azerbaijan, West Azerbaijan, Ilam, Kurdistan, Hamedan, and Lorestan (*n*=4138) [86](Tables 1 and 2).

Among the 78 included studies, 75 studies reported only CE-infected individuals, 1 study reported only AE-infected patients [90], and two studies reported both AE and CE-infected cases [73, 74]. With regard to the few numbers of AE studies in Iran, only three studies were included in the systematic review, and two of them [73, 74] were assessed in the meta-analysis.

**Fig. 2:**
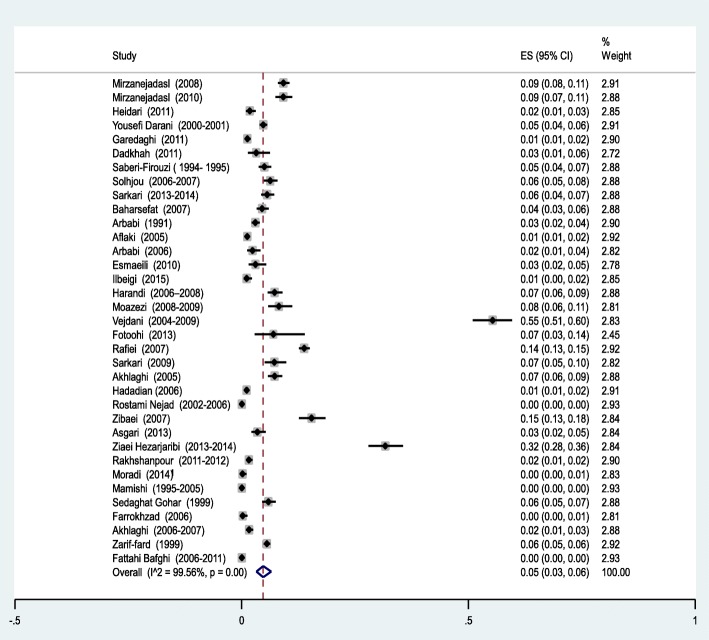
Forest plot diagram of the total prevalence of cystic echinococcosis (CE) in Iran. The middle point of each line indicates the prevalence rate and the length of line indicates 95% confidence interval of each study. The opened diamond is representatives of the overall prevalence of the studies

According to the results of the meta-analysis, the pooled prevalence of CE in Iran was estimated at 5% [95% CI: 3-6%] (Fig. 2). In most studies (31 out of 77 studies, 40%), CE-infected cases were in the age group of 20-40 years. Housewives were the most affected group by hydatidosis (*n*=24/77, 31%), followed by illiterate people (*n*=11/77, 14%) and farmers (*n*= 9/77, 12%). In children, boys were more infected than girls (*n*=315/548, 57.5%). Serological techniques (*n*=27/77, 35%) were the most utilized method for diagnosis of CE, followed by clinical and radiographic methods (*n*=21/77, 27%), and the combination of methods (i.e. serological, clinical and radiographic methods) (*n*=14/77, 18%) (Table 1).

**Fig. 3:**
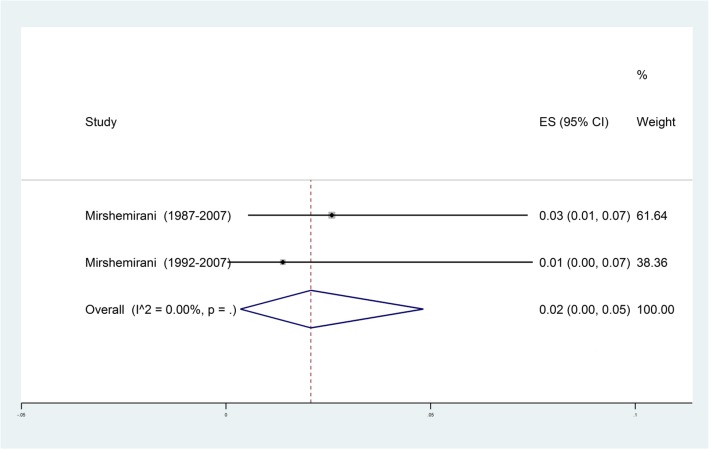
Forest plot diagram of the total prevalence of alveolar echinococcosis (AE) in Iran. The middle point of each line indicates the prevalence rate and the length of line indicates 95% confidence interval of each study. The opened diamond is representatives of the overall prevalence of the studies

Results of meta-analysis demonstrated a pooled prevalence of (2%, 95% CI: 0-5%) for AE in Iran (Fig. 3). In overall, a total of 22 cases of AE were reported in 3 included studies. Two studies were conducted on children from pediatric hospitals of Tehran [73, 74], but they did not mention the exact age range and gender of AE patients. The other study was performed on AE patients from Khorasan Razavi province (North-east of Iran), and most of them were women with a mean age of 46.11±15.14 years. Clinical and imaging methods (i.e. ultrasonography, CT scan, and MRI) were the most used diagnostic methods for AE (Table 2).

**Fig. 4:**
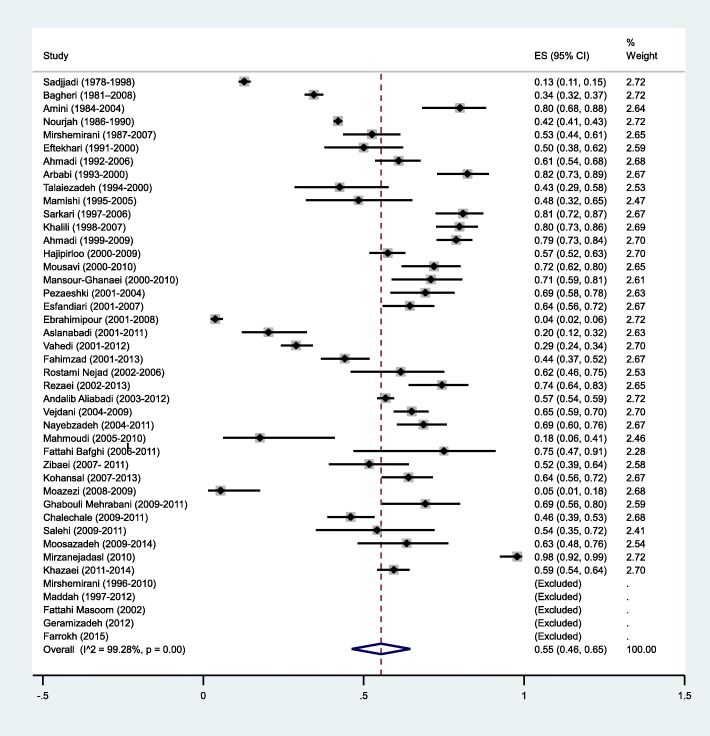
Forest plot diagram of the total prevalence of hydatid cyst in liver. The middle point of each line indicates the prevalence rate and the length of line indicates 95% confidence interval of each study. The opened diamond is representatives of the overall prevalence of the studies

**Fig. 5:**
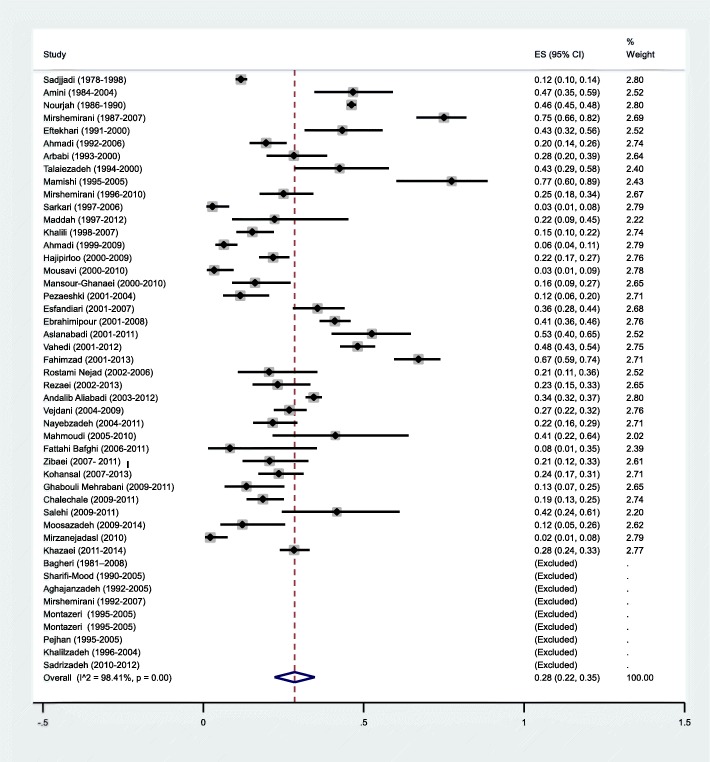
Forest plot diagram of the total prevalence of hydatid cyst in the lung. The middle point of each line indicates the prevalence rate and the length of line indicates 95% confidence interval of each study. The opened diamond is representatives of the overall prevalence of the studies

Different sites of hydatid cyst formation are shown in Table 3 and the pooled prevalence of hydatid cysts in different parts of the body are listed in Table 4. According to the results, liver [55%, 95% CI: 46-65%] and lung [28%, 95% CI: 22-35%] were the most common sites of cyst formation (Figs. 4 and 5). The organs, in which the total number of cysts was less than 10, were considered as unusual site of cyst formation, including: heart (*n*=8, 0.06%), spinal cord (*n*=7, 0.05%), bone (*n*=6, 0.04%), intestine (*n*=6, 0.04%), diaphragm (*n*=5, 0.03%), bladder (*n*=5, 0.03%), ovary (*n*=5, 0.03%), pleura (*n*=5, 0.03%), bronchus (*n*=5, 0.03%), mediastinum (*n*=5, 0.03%), chest wall (*n*=4, 0.03%), mesentrium (*n*=3, 0.02%), adrenal glands (*n*=3, 0.02%), femur (*n*=3, 0.02%), uterus (*n*=3, 0.02%), esophagus (*n*=2, 0.01%), pericardium (*n*=2, 0.01%), facial sinuses (*n*=2, 0.01%), prostate (*n*=2, 0.01%), breast (*n*=2, 0.01%), inguinal canal (*n*=2, 0.01%), choledochus (*n*=2, 0.01%), colon (*n*=2, 0.01%), muscle (*n*=2, 0.01%), CNS (*n*=2, 0.01%), axillary cavity (*n*=1, 0.007%), skin (*n*=1, 0.007%), vertebrate (*n*=1, 0.007%), sub-cutaneous (*n*=1, 0.007%), neck (*n*=1, 0.007%), buttock (*n*=1, 0.007%), leg (*n*=1, 0.007%), bilious canal (*n*=1, 0.007%), supraclavicular area (*n*=1, 0.007%), and supra-pubic (*n*=1, 0.007%).

**Table 3:** Location of cysts in patients with cystic echinococcosis (CE)

First author	CE positive	Liver		Lung		Spleen		Kidney		Brain		Peritoneum		Pelvis		Gallbladder		Abdomen		Pancreas		Retro-peritoneal		Unusual cyst formation cite		Multiple organ involvement	
		N	%	N	%	N	%	N	%	N	%	N	%	N	%	N	%	N	%	N	%	N	%	N	%	N	%
Sadjjadi [31]	1227	157	13	144	12													12	1					7	0.6		
Bagheri [47]	1024	353	34	1024	100	28	3															12	1				
Amini [71]	60	48	80	28	47																						
Nourjah [72]	4850	2037	42	2241	46					131	3							102	2								
Mirshemirani [73]	116	61	39	87	55	2	1	2	1					2	1			2	1					1	0.6		
Sharifi-Mood [69]	49			49	100																					2	4
Eftekhari [42]	60	30	50	26	43			1	2															3	5		
Aghajanzadeh [3]	152			152	100																					30	20
Ahmadi [37]	179	109	60.9	35	20	5	3	5	3	1	0.6	4	2			2	1			1	0.6			5	3	1	0.6
Ahmadi [38]	24					5	21	5	21	1	4	4	17			2	8			1	4			5	21	1	4
Mirshemirani [74]	72			72	100																					10	14
Arbabi [40]	85	70	85	24	28	2	2	2	2			3	4							1	1			3	3.5		
Talaiezadeh [53]	40	17	43	17	43																					6	15
Montazeri [26]	383			383	100																					107	28
Montazeri [26]	62			62	100																					10	16
Pejhan [75]	82			82	100																					15	18
Mamishi [76]	31	15	48	24	77																					11	35
Khalilzadeh [77]	11			11	100																						
Mirshemirani [78]	100	100	100	25	25																						
Sarkari [55]	105	85	81	3	3			3	3															2	2	10	10
Maddah [90]	18	18	100	4	22																						
Khalili [24]	144	115	80	22	15			2	1			1	0.7											1	0.7		
Ahmadi [80]	203	160	79	13	6			4	2	4	2	1	0.5	3	1.5			1	0.5					7	3	10	5
Hajipirloo [87]	294	169	57.5	64	22	7	2	9	3	7	2																
Mousavi [81]	89	64	81	3	3	1	1	1	1	2	2			3	3									3	3		
Mansour-Ghanaei [35]	62	44	77	10	16			1	2							1	2			1	2			3	5	2	3
Pezaeshki [82]	78	54	69	9	11.5	6	8	1	1			1	1					3	4	1	1			3	4		
Esfandiari [63]	132	85	64	47	35.5																						
Ebrahimipour [48]	400	15	38	164	41																					51	13
Aslanabadi [20]	59	12	20	31	53			4	7			1	2	1	2			1	2							9	15
Vahedi [27]	318	92	29	153	48	7	2	7	2	2	0.6			1	0.3	11	3	5	2	2	0.6			40	13		
Fahimzad [41]	161	71	44	108	67	4	3	4	3	3	2													4	2.5		
Rostami Nejad [59]	39	24	61.5	8	20.5			1	3	2	5	1	3	1	3									3	8		
Rezaei [67]	82	61	74	19	23	1	1			1	1																
Andalib Aliabadi [49]	1342	763	57	462	34	5	0.4	16	1	7	0.5			1	0.07					1	0.1			10	0.7		
Vejdani [45]	291	189	65	78	27	2	0.7	2	0.7			2	0.7	5	2	2	0.7			2	0.7			9	2	3	1
Nayebzadeh [60]	134	92	69	29	22	1	0.7	2	1.5	2	1.5													1	0.7	7	5
Mahmoudi [83]	17	3	18	7	41					3	18															8	47
Fattahi Bafghi [88]	12	9	75	1	33					1	33													1	33		
Zibaei [61]	58	30	52	12	21			1	2	1	2	11	19	1	2									1	2	1	2
Kohansal [89]	136	87	64	32	23.5	2	1.5	1	0.7					2	1.5											13	9.5
Moazezi [44]	37	2	0.4																								
Ghabouli Mehrabani [28]	52	36	60	7	13	1	2					2	4													6	11.5
Chalechale [46]	172	79	60	32	24	3	2			2	1.5	1	0.7	1	0.7			1	0.7							13	7.5
Salehi [66]	24	13	54	10	42																					1	4
Moosazadeh [64]	41	26	63	5	12					1	2							1	2							1	2
Mirzanejadasl [22]	92	90	98	2	2																						
Sadrizadeh [50]	87			87	100																					17	20
Khazaei [51]	357	212	59	101	28	3	0.8	2	0.6									7	2					2	0.5	30	8

**Table 4:** Sub- group analysis of the prevalence of cystic echinococcosis (CE) in different sites of body

Site of cyst formation	Prevalence (95% CI)	*I*^*2*^ (%)	Heterogeneity (χ^2^)	*P* value
Liver	0.55 (0.46-0.65)	99.28	5169.09	< 0.001
Lung	0.28 (0.22-0.35)	98.41	2334.25	< 0.001
Spleen	0.02 (0.01-0.02)	62.98	45.92	< 0.001
Kidney	0.01 (0.01-0.02)	9.5	23.21	0.33
Brain	0.02 (0.01-0.02)	75.08	64.22	< 0.001
Peritoneum	0.01 (0.00-0.02)	52.25	23.03	0.02
Pelvis	0.01 (0.00-0.01)	42.24	17.3	0.07
Gallbladder	0.02 (0.00-0.03)	47.72	7.65	0.11
Abdomen	0.01 (0.01-0.02)	55.8	20.36	0.02
Pancreas	0.00 (-0.00-0.00)	2.57	7.18	0.41
Unusual localized cyst	0.02 (0.01-0.03)	73.39	75.16	< 0.001
Multiple organ involvement	0.1 (0.07-0.12)	91.96	298.39	< 0.001

**Fig. 6:**
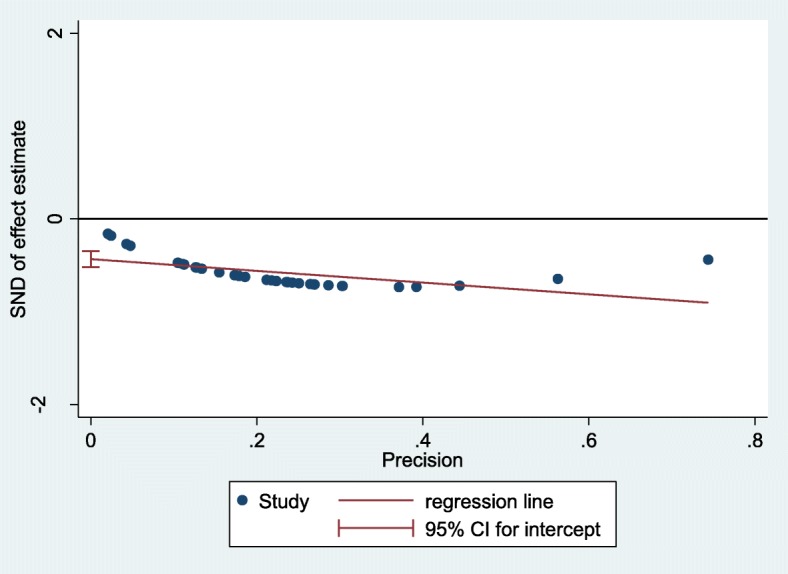
The Egger’s test graph to test for publication bias (*P* value < 0.0001)

Results revealed a strong heterogeneity (*I*^*2*^ = 99.56%; *P*<0.001) among the selected studies (Fig. 2). The subgroup analysis was performed (Table 5) and its results illustrated that the prevalence of CE was significantly higher in the North [9%, 95% CI: 4-18%] and West [6%, 95% CI: 3-11%] regions of Iran. It was significantly more prevalent in patients younger than 40 years of age [7%, 95% CI: 4-12%] and was lower in urban dwellers [1%, 95% CI: 0-5%]. There were statistically significant differences between the overall prevalence of CE in the subgroup that used the combination of serological, clinical and imaging diagnostic methods [7%, 95% CI: 5-9%] and subgroup that used only serological techniques [4%, 95% CI: 3-6%]. There were no significant differences in the prevalence of CE in the subgroups with low and high quality (*P*= 0.48). Based on the results of Egger’s regression test (Fig. 6) the publication bias among included studies could not be ignored (*P* < 0.0001).

**Table 5:** Sub-group meta-analysis of the prevalence of cystic echinococcosis (CE) in Iran

Subgroup variable		Prevalence (95% CI)	*I*^*2*^ (%)	Heterogeneity (χ^2^)	*P* value	Interaction test (χ^2^)	*P* value
Geographical distribution	North	0.09 (0.04-0.18)	98.73	315.38	< 0.001	12.95	< 0.001
	Center	0.02 (0.01-0.03)	98.87	970.4	< 0.001		
	West	0.06 (0.03-0.11)	99.71	4477.29	< 0.001		
	East	0.05 (0.01-0.11)	96.4	83.22	< 0.001		
Age	< 40 years	0.07 (0.04-0.12)	99.23	1937.99	< 0.001	40.03	< 0.001
	≥ 40 years	0.05 (0.03-0.06)	95.24	230.86	< 0.001		
	ND ^a^	0.01 (0.00-0.01)	98.8	465	< 0.001		
Quality score	Low	0.05 (0.01-0.12)	99.59	2426.9	< 0.001	0.49	0.48
	High	0.04 (0.03-0.06)	99.56	5225.56	< 0.001		
Diagnostic lab method	Serological	0.04 (0.03-0.06)	98.22	1459.33	< 0.001	257.4	< 0.001
	Serological, clinical, and imaging^b^	0.07 (0.05-0.09)	79.06	14.33	< 0.001		
Place of residence	Urban	0.01 (0.00-0.05)	98.48	262.31	< 0.001	18.63	< 0.001
	Urban and rural	0.06 (0.03-0.1)	99.57	-	-		
	Rural and nomads	0.06 (0.02-0.12)	98.13	214.1	< 0.001		
	ND	0.05 (0.01-0.12)	99.66	1191.6	< 0.001		
All studies		0.05 (0.00-0.16)	99.56	7797.5	< 0.001	-	-

^a^ *ND* Not-determined

^b^Clinical and imaging methods are included: clinical manifestations of the patients, in combination with the results of imaging and radiographic diagnostic methods (computed tomography (CT) scan, magnetic resonance imaging (MRI), chest-X ray, ultrasonography, bronchoscopy, or radiology) or pathological and histopathological examinations

## Discussion

According to our knowledge, this is the third systematic review and meta-analysis about the prevalence of human echinococcosis in Iran. In the first study [2], Shafiei and colleagues evaluated the seroprevalence of human CE in Iran during the time period of 1985 to 2015. They assessed only articles which diagnosed the disease based on serological techniques and did not include other studies that used clinical, imaging, and pathological diagnostic methods. In the second study [9], Khalkhali *et al*., studied the prevalence of both animal and human hydatidosis in Iran (from January 1990 to December 2015), with the most focus on definitive and intermediate hosts. Both studies did not evaluate the situation of AE in Iran and distribution of infection in different parts of the body. Thus, we performed this study to have a more comprehensive assessment about echinococcosis in Iran.

The result of our study demonstrated that the estimated pooled prevalence of CE in Iran during a period of 27 years (from January 1990 to August 2017) was 5% [95% CI: 3-6%]. This was the same as the rate reported by Shafiei *et al*., [5%, 95% CI: 4-6%] [2] and similar to the rate of Khalkhali *et al*., [4.2%, 95% CI: 3–5.5%] and was higher than the rate reported by Li *et al*., (3.2%) from China [94] and Solomon *et al*., (3.8%) from Kenya [95]. The differences in the prevalence of CE in various studies can be due to the differences in climatic conditions in each region, which could affect the viability of parasite’s eggs, the frequency of infected final hosts and livestock farming in each region, level of contact with dogs, and occupation of the understudy population [46].

As mentioned before, the information about AE in Iran is so limited and there are few reports in this field. This is because that in contrast to the CE, AE is a rare infection in Iran [76]. In the study conducted by Wacław *et al*., from Poland [96], 120 cases of AE-infected patients were reported during a 20 years study, suggesting that AE is an emerging infection in this country. . Although, AE is an uncommon infection in Iran, due to the high lethality of this disease, physicians should consider the possibility of AE in the differential diagnosis when dealing with patients that have extensive, infiltrating tumor-like lesions in the liver [97, 98].

Our investigations revealed that in adult group, the echinococcosis was more prevalent among women, especially housewives. A reversed result was reported by Conchedda *et al*., from Italy [99], which the patients’ male to female ratio was 1.36. It seems that in some areas of Iran especially in villages, women have more contact with sources of infection (i.e. soil, dogs, and contaminated raw vegetables) than men [46, 62]. They participate in farming and herding and have contact with domestic animals (intermediate hosts of *E. granulosus*) when feed them or clean their living space or when milking [24, 28, 43]. which is mostly common among pregnant women.

Assessment of organ distribution of cysts demonstrated that different organs were involved in CE (Tables 3 and 4), however, liver was the most affected organ [55%, 95% CI: 46-65%]. This was in agreement with other studies [99–101]. . This observation can be explained by the fact that the liver and lungs are the most important body filters and are the first sites to encounter the migrating parasite larvae, and usually a few parasites can escape from them and gain access to other organs [102].

The meta-analysis revealed that the prevalence of CE was higher in the North [9%, 95% CI: 4-18%] and West [6%, 95% CI: 3-11%] parts of Iran. The highest rate of CE was reported from Kermanshah (a western province of Iran) [55%, 95% CI: 51-60%] and Mazandaran (a northern province of Iran) [32%, 95% CI: 28-36%] and the lowest rate was related to Lorestan, Yazd, and Sistan and Baluchestan (<1%). This was in contrast to the results of Shafiei *et al*., [2] and Khalkhali *et al*., [9] that the highest rates were related to the West and Southwest of Iran. A possible explanation for this controversy can be the different studies that were evaluated in the two other systematic reviews than our study.

Our findings showed that patients in the age group of 20-40 years [7%, 95% CI: 4-12%] were the main sufferers of CE. In other studies from Turkey [103] and Italy [99] CE was more prevalent among middle-aged and old patients. Since the 20-40 years people are among the most active age groups of the society, CE can have devastating economic damages [71, 81].

Our study revealed a higher CE prevalence rate among rural dwellers and nomads [6%, 95% CI: 2-12%] than city residents [1%, 95% CI: 0-5%]. Similar results were reported from other studies [100, 104, 105]. This is due to the lifestyle and occupation of villagers and nomads that result in their higher exposure to the infection sources [61]. Serological techniques (*n*=27/77, 35%) were the most frequently used methods for diagnosis of CE. However, higher rates of infection were detected by the combination of serological, clinical, and imaging methods [7%, 95% CI: 5-9%] compared to the serologic methods alone [4%, 95% CI: 3-6%). Investigations revealed that the positivity of individuals with serological methods is not indicative of the definite involvement of a person in CE or the presence of active hydatid cysts in the body [29]. These techniques have some limitations; therefore, serodiagnostic tests should be used as complementary or confirmatory methods of CE detection and a combination of serologic, clinical, and imaging approaches is the most appropriate CE-diagnostic method.

There are some limitations to this study. First, most of the hospital-based retrospective studies reported the number of infected cases during a specific time interval, but did not report the prevalence rate of infection. Therefore, these studies did not include into the meta-analysis. This means that some of the potential useful studies were excluded and their data were not utilized. Second, there were limited data from some provinces of Iran such as Golestan, Ilam, Markazi, West Azerbaijan, Yazd, and Zanjan.

## Conclusion

In conclusion, given to the importance of echinococcosis on human health and domestic animals industry, it is necessary to implement monitoring and control measures in this regard. This requires public health education and awareness about the dangers of the disease and its transmission and preventive routes, education on the appropriate ways of washing and disinfecting of vegetables and fruits, education on the correct ways of animal slaughtering, prevention on feeding dogs by viscera of home-slaughtered animals, prevention on direct contact by dogs’ feces, enforce legislation on meat inspection and improve veterinary services, fighting stray dogs, treating and vaccination of dogs and domestic animals, investigation on the pollution of water and soil resources in endemic areas such as the North and West of Iran in terms of *Echinococcosis*’ eggs.

## Data Availability

All data obtained

## Abbreviations

AE: Alveolar echinococcosis
CE: Cystic echinococcosis
CIE: Counterimmunoelectrophoresis
CT: Computed tomography scan
ELISA: Enzyme-linked immunosorbent assay
IFA: Indirect fluorescent antibody test
IHA: Indirect hemagglutination antibody test
MRI: Magnetic resonance imaging
WHO: World Health Organization
